# Cytotoxic effects of the compound *cis*-tetraammine(oxalato)ruthenium(III) dithionate on K-562 human chronic myelogenous leukemia cells

**DOI:** 10.1186/2193-1801-3-301

**Published:** 2014-06-19

**Authors:** Flávia de Castro Pereira, Aliny Pereira de Lima, Cesar Augusto Sam Tiago Vilanova-Costa, Wanessa Carvalho Pires, Alessandra de Santana Braga Barbosa Ribeiro, Lucas Carlos Gomes Pereira, Luiz Alfredo Pavanin, Wagner Batista dos Santos, Elisângela de Paula Silveira-Lacerda

**Affiliations:** Laboratório de Genética Molecular e Citogenética, Instituto de Ciências Biológicas, Universidade Federal de Goiás - UFG, Campus Samambaia (Campus II), Cx. Postal: 131, Goiânia, GO 74001-970 Brazil; Instituto de Química, Universidade Federal de Uberlândia - UFU, Uberlândia, MG 38400-902 Brazil; Instituto de Ciências Exatas e da Terra, Universidade Federal de Mato Grosso - UFMT, Barra do Garças, MT 78698-000 Brazil

**Keywords:** *cis*-tetraammine(oxalato)ruthenium(III) dithionate, Cytotoxic activity, K-562, Ruthenium(III) compounds, Immunomodulatory activity, Apoptosis

## Abstract

Chemotherapy is a common treatment for leukemia. Ruthenium complexes have shown potential utility in chemotherapy and photodynamic therapy. The identification of new chemotherapeutics agents is critical for further progress in the treatment of leukemia. Ruthenium complexes generally have lower toxicities compared to cisplatin attributed to their specific accumulation in cancer tissues. Based on these evidences, in the present work we studied the cytotoxic activity of the ruthenium(III) compound *cis*-tetraammine(oxalato)ruthenium(III) dithionate - {*cis*-[Ru(C_2_O_4_)(NH_3_)_4_]_2_(S_2_O_6_)} against human chronic myelogenous leukemia cells (K-562) tumor cell line. The tested compound induces cell death in a dose and time dependent manner on K-562 cells. It is found that the effect was improved linearly while prolonging the incubation time. Compared to the cell cycle profiles of untreated cells, flow cytometric analysis indicated the sub-G1 arresting effect of ruthenium compound on K-562 cells. In our study, {*cis*-[Ru(C_2_O_4_)(NH_3_)_4_]_2_(S_2_O_6_)} shows a significant increase in tailed cells in any of the concentrations tested compared with negative control. Consequently, the concentration of {*cis*-[Ru(C_2_O_4_)(NH_3_)_4_]_2_(S_2_O_6_)} might be associated cytotoxicity with direct effect on K-562 cells DNA. Thus, it can be deducted that ruthenium-based compounds present selectivity to enter both tumor and normal cells. Additional studies are needed to determine the molecular mechanisms of the active components and to evaluate the potential in vivo anticancer activity of the *cis*-tetraammine(oxalato)ruthenium(III) dithionate.

## 1. Introduction

Leukemia is a major type of cancer affecting a significant segment of the population, and especially children. In fact, leukemia is the most frequent childhood cancer, with 26% of all cases, and 20% mortality (Canadian Cancer Society/National Cancer Institute of Canada 2005). The American Cancer Society (ACS) estimated that 47,150 new cases of leukemia would be diagnosed in the United States in 2012, whereas about 23,540 adults and children would die of leukemia during 2012 (American Cancer Society 2009).

Although the incidence rate for this disease remains relatively unchanged, some success has fortunately been attained in its treatment. But even if the success of clinical trials in identifying new agents and treatment modalities has been significant, current treatments have many limitations related to their side effects and the development of acquired drug resistance (Robert and Jarry 2003) The new therapeutic agents thus needed should be more active and produce less side effects and they also should act through a mechanism different from that of cytotoxic agents already used (Menezes et al. 2007; Silveira-Lacerda et al. 2009).

Chemotherapy is a common treatment for leukemia (Ge et al. 2009). In general the therapy uses a number of different anticancer drugs, which destroy cancer cells by preventing them from growing and dividing rapidly. Unfortunately, a number of the body’s normal, non-cancerous cells (e.g., hair cells, red and white blood cells, blood-clotting platelets, and cells of the gastrointestinal mucosa) also divide rapidly and are harmed by chemotherapy (Ge et al. 2009; Kim et al. 2002). The side effects of chemotherapy hamper many normal activities of patients undergoing treatment (Kim et al. 2002).

The preparations of metallo complexes with potential antitumor activity has been one of the main targets of transition metal chemistry since Rosenberg’s discovery of cisplatin {*cis*-diamminedichloridoplatinum(II), *cis*-[Pt(NH_3_)_2_Cl_2_]} cytotoxic activity in the 1960s (Rosenberg et al. 1965). In 1978, cisplatin was approved as the first platinum based drug for the oncology treatment, although several negative side-effects (nephrotoxicity, neurotoxicity, nausea, etc.) had been induced on treated patients (Kelland and Farrell 2000). Nevertheless, cisplatin was followed by carboplatin {*cis*-diammine-1,1′ -cyclobutanedicarboxylateplatinum(II), [Pt(NH_3_)_2_(cbdc)], approved in 1985} and oxaliplatin {1R,2R-diamminocyclohexaneoxalatoplatinum(II), [Pt(dach)(ox)], approved in 1996}, which met requirements of improving antitumor activity and reducing disadvantages of cisplatin, carboplatin and oxaliplatin represent the second, and third platinum-based drug generations, respectively ((Kelland and Farrell 2000; Štarha et al. 2009). Nowadays, not only platinum-bearing complexes are extensively studied with the aim to broaden a spectrum of transition metal-based complexes which could be used in the treatment of cancer (Štarha et al. 2009).

In addition to the other metal complexes cisplatin have been developed using heavy metals. For example, gold complexes have been developed for the treatment of rheumatoid arthritis, silver complexes as anti-microbial agents, antimony complexes for the treatment of leishmaniasis, vanadium(IV) complexes as antiviral and antidiabetic agents, arsenic trioxide (Trisenox) for the treatment of acute promyelocytic leukaemia, and metal-activated bleomycin for the treatment of Hodgkin’s lymphoma and testicular cancer. Transition-metal-based therapeutic agents currently under clinical trials include third generation antitumor platinum complexes such as liposomal cisplatin (Lipoplatin), satraplatin, and picoplatin, the antimalarial ferrocene–quinoline conjugate ferroquine (Lung et al. 2013).

Rhodium and Iridium has also been used for the synthesis of these metal complexes, as they allow greater selectivity and affinity molecules. The iridium (III) already shows results inhibiting TNF-α in HepG2 line through a hydrophobic binding (Meggers 2009; Leung et al. 2012). These metal complexes possess many advantages that make them suitable for the development of new therapeutic agents and compounds of ruthenium has been highlighted because ruthenium complexes have shown potential utility in chemotherapy and photodynamic therapy (Clarke 2003; Ronconi and Sadler 2007). Ruthenium complexes generally have lower toxicities compared to cisplatin attributed to their specific accumulation in cancer tissues (Sava et al. 1998). *In vitro* and in vivo studies show high anticancer activity of ruthenium complexes and some of them are currently undergoing clinical trials (Jakupec et al. 2005; Hartinger et al. 2006).

The identification of new chemotherapeutics agents is critical for further progress in the treatment of leukemia. In comparison to the platinum(II) antitumor complexes currently used in the clinic, ruthenium compounds offer potentially reduced toxicity, a novel mechanism of action, the prospect of non-cross-resistance, and a different spectrum of activity (Clarke 2003). The reduced toxicity is in part due to the ability of ruthenium complexes to mimic the binding of iron to molecules of biological significance, exploiting the mechanisms that the body has evolved for non-toxic transport of iron (Frasca et al. 2001). This reduced toxicity, together with non-cross-resistance in cisplatin-resistant cancer cells, is particularly attractive attributes of these complexes (Allardyce et al. 2003).

Based on these evidences, in the present work we studied the cytotoxic activity of the ruthenium(III) compound *cis*-tetraammine(oxalato)ruthenium(III) dithionate {*cis*-[Ru(C_2_O_4_)(NH_3_)_4_]_2_(S_2_O_6_)} against human chronic myelogenous leukemia (K-562) tumor cell line.

## 2. Results

### 2.1 The *cis*-[Ru(C_2_O_4_)(NH_3_)_4_]_2_(S_2_O_6_) compound reduces viability of K-562 cells

**Figure 1:**
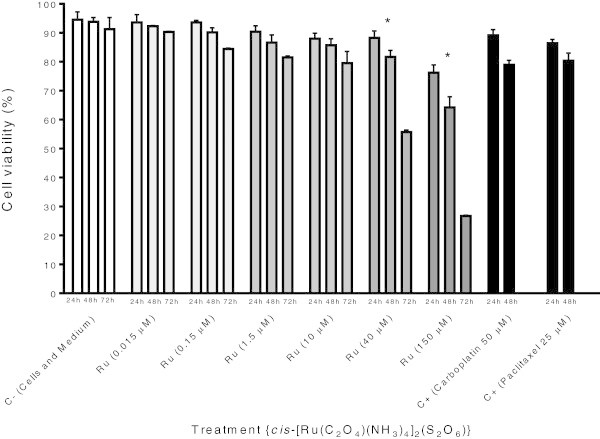
**Cytotoxic activity of** ***cis*** **-tetraammine(oxalato)ruthenium(III) dithionate compound towards K-562 cell line.** The data show the mean ± S.D. (standard deviation) of three independent experiments [GraphPad Prism version 4.02 for Windows (GraphPad Software, San Diego, California, USA)]. *p < 0.05 vs. negative control.

Results derived from Trypan blue staining essay revealed that K-562 cells cultured with concentrations 40 and 150 μM of ruthenium(III) compound showed significant reduction of proliferation after 72 h of exposition, with viabilities ranging from 88.2% to 55.6% when treated with 40 μM for 24 and 72 h; and 76.2% to 26.7% when treated with 150 μM for 24 and 72 h Figure 1.

### 2.2 The *cis*-[Ru(C_2_O_4_)(NH_3_)_4_]_2_(S_2_O_6_) compound presents cytotoxic activity towards K-562 tumor cell lines

**Figure 2:**
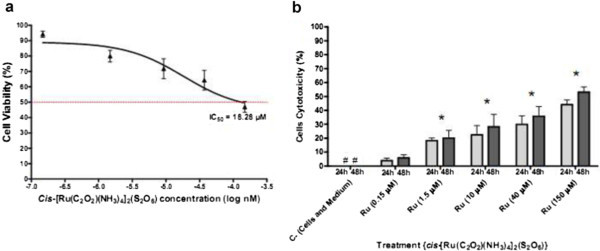
**Anti-proliferative activity of** ***cis*** **-tetraammine(oxalato)ruthenium(III) dithionate compound towards K-562 cell line (a and b).** The data show the mean ± S.D. (standard deviation) of three independent experiments [GraphPad Prism version 4.02 for Windows (GraphPad Software, San Diego, California, USA)]. *p < 0.05 vs. negative control. # = 0%. Dotted line = IC_50_ concentration for 48 h of treament.

To verify the cytotoxic activity of *cis*-[Ru(C_2_O_4_)(NH_3_)_4_]_2_(S_2_O_6_) on K-562 cell lines, tumor cells were cultured for 24 and 48 h in the presence of different concentrations of ruthenium compound. MTT reduction assay revealed that ruthenium compound produced concentration and time-dependent cytotoxicity effects on K-562 cells. The results Figure 2b show that the ruthenium(III) compound induced low [22.4% (24 h) to 28.2% (48 h) and 29.8% (24 h) to 35.7% (48 h) for concentrations 10 and 40 μM, respectively] to up [44% (24 h) and 53% (48 h) for concentration 150 μM] of cytotoxic activity against K-562. After incubation for 48 h, the IC_50_ value was 18.28 μM (Figure 2a).

### 2.3 The *cis*-[Ru(C_2_O_4_)(NH_3_)_4_]_2_(S_2_O_6_) induced apoptosis in K-562 cells as verified by DNA ladder analysis

The significantly reduced number of K-562 cells in the presence of *cis*-tetraammine(oxalato)ruthenium(III) dithionate on previous assays suggests that this compound could be inducing cell death via apoptosis. To examine pro-apoptotic properties of the ruthenium(III) compound, K-562 cells were cultured in the presence of different concentrations of ruthenium compound (from 0.15–150 μM) and then DNA ladder analysis was performed via agarose gel electrophoresis. K-562 cells cultured in the presence of ruthenium compound but not in medium alone presented DNA fragmentation (Data not shown).

### 2.4 The *cis*-[Ru(C_2_O_4_)(NH_3_)_4_]_2_(S_2_O_6_) presents genotoxic effects against K-562 tumor cells

**Figure 3:**
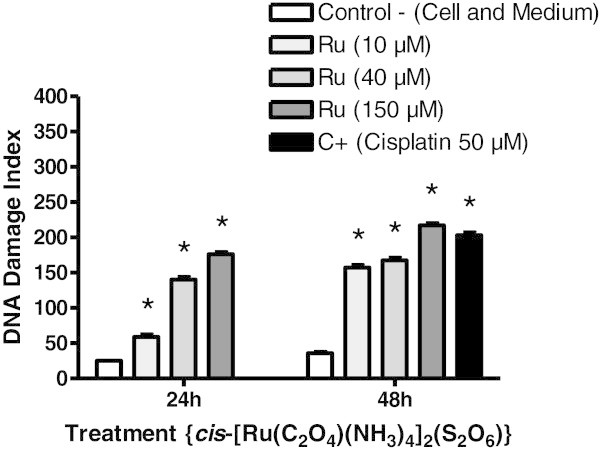
**Induction of DNA strand breaks of K-562 cells cultured in the presence of** ***cis*** **-[Ru(C**_**2**_ **O**_**4**_ **)(NH**_**3**_ **)**_**4**_ **]**_**2**_ **(S**_**2**_ **O**_**6**_ **) compound.** Results in the figure represent the mean ± S.D. of 3 independent experiments using triplicate samples. *Increased above control at p < 0.05.

The alkaline comet assay has been used to assess the possible genotoxicity of *cis*-tetraammine(oxalato)ruthenium(III) dithionate against K-562 cells. Results indicate that K-562 tumor cells cultured with ruthenium(III) complex show a significant increase in DNA damage index in any of the concentrations tested compared with negative control, in a dose-dependent manner (Figure 3). Results show an average DNA damage index of 59 for 10 μM, 140 for 40 μM, 176 for 150 μM when K-562 tumor cells were exposed to ruthenium(III) for 24 h; and 108 for 10 μM, 154 for 40 μM and 197 for 150 μM, for 48 h of exposition. Thus, the concentration of *cis*-[Ru(C_2_O_4_)(NH_3_)_4_]_2_(S_2_O_6_) might be associated cytotoxicity with direct effect on K-562 cells DNA (p < 0.05).

### 2.5 The *cis*-[Ru(C_2_O_4_)(NH_3_)_4_]_2_(S_2_O_6_) increased the number of K-562 tumor cells in sub-G1 phase, an indicative of apoptosis

**Figure 4:**
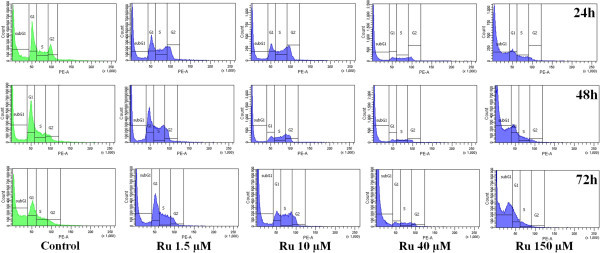
**Cell cycle profile histogram of K-562 cells treated with** ***cis-*** **tetraammine(oxalato)ruthenium(III) dithionate.** PI fluorescence was analyzed by flow cytometry (FACS Canto II, BD Biosciences, San José, CA, USA).

The cell cycle distribution of K-562 cells treated with different concentrations of ruthenium compound (from 1.5–150 μM) for 24, 48 and 72 h revealed a prominent increasing of cells on sub-G1 phase at concentration 40 and 150 μM and reduction on G0/1, S and G2/M phases. Figure 4 shows that treatment with 40 and 150 μM of *cis*-[Ru(C_2_O_4_)(NH_3_)_4_]_2_(S_2_O_6_) for 24, 48 and 72 h caused an increase in the proportion of cells in the sub-G1-peak correlating with fewer cells in G0/G1, S and G2/M. The fraction of sub-G1 cells increased from 14.5% in the control to 59% at 24 h; 10.7% in the control to 68.6% at 48 h; 15.5% in the control to 84.7% for 72 h on cells treated with 40 μM of *cis*-tetraammine(oxalato)ruthenium(III) dithionate. The same effect was observed when K-562 tumor cells were treated with 150 μM of ruthenium(III) compound. The ruthenium(III) compound caused a minor S phase accumulation after incubation at concentrations of 40 and 150 μM for all periods of exposition Table 1.

**Table 1:** **Cell cycle analysis of K-562 tumor cell lines after treatment with** ***cis*** **-tetraammine(oxalato)ruthenium(III) dithionate**

	Control	Ru 1.5 μM	Ru 10 μM	Ru 40 μM	Ru 150 μM
**24 h**	**Mean ± S.D.**	**Mean ± S.D**	**Mean ± S.D**	**Mean ± S.D**	**Mean ± S.D**
**Sub-G0/G1**	14.45 ± 0.6	30.20 ± 2.1	31.20 ± 1.4	59.00 ± 2.3	68.90 ± 1.8
**G1**	27.20 ± 0.3	25.25 ± 2.1	19.55 ± 0.9	11.50 ± 1.7	14.75 ± 0.9
**S**	19.05 ± 0.1	27.50 ± 1.1	29.10 ± 1.1	18.60 ± 2.1	11.40 ± 0.8
**G2**	14.10 ± 0.1	14.85 ± 0.8	18.10 ± 0.7	9.15 ± 1.6	4.00 ± 0.1
**48 h**					
**Sub-G0/G1**	10.70 ± 0.7	24.65 ± 1.1	40.55 ± 2.6	68.60 ± 1.1	67.60 ± 0.1
**G1**	35.65 ± 0.6	31.70 ± 1.0	14.85 ± 2.6	8.35 ± 0.4	19.30 ± 0.4
**S**	21.35 ± 0.4	30.05 ± 0.8	26.35 ± 2.1	14.35 ± 0.9	9.75 ± 0.2
**G2**	10.00 ± 1.4	12.50 ± 0.3	17.05 ± 2.3	7.85 ± 0.1	2.95 ± 0.1
**72 h**					
**Sub-G0/G1**	15.50 ± 16.0	34.50 ± 1.4	45.95 ± 1.1	84.70 ± 0.3	70.50 ± 2.8
**G1**	35.45 ± 10.1	31.75 ± 0.5	17.05 ± 0.2	4.95 ± 0.1	18.70 ± 1.0
**S**	20.05 ± 4.7	23.80 ± 0.6	26.05 ± 0.6	7.20 ± 0.6	9.25 ± 1.6
**G2**	7.45 ± 0.8	9.30 ± 0.4	10.50 ± 0.3	3.10 ± 0.1	1.45 ± 0.2

The percentage of cells in G0/G1, S, G2/M and sub-G1 was analyzed using ModFit LT software (Verity Software House, Topsham, ME, USA). Percentage of viable cells at each phase of the cell cycle (G0/G1, S, G2/M). Data represent mean values ± SD of triplicate samples or a histogram for each condition and cell type. The results shown are representative of at least three similar experiments. *p < 0.05 vs. control.

## 3 Discussion

Apoptosis is an active physiological process resulting in cellular self-destruction that involves specific morphological and biochemical changes in the nucleus and cytoplasm (Kostova 2006). Agents that suppress the proliferation of malignant cells by inducing apoptosis may represent a useful mechanistic approach to both cancer chemoprevention and chemotherapy. While many anticancer agents have been developed, unfavourable side effects and resistance are serious problems (Panchal 1998). Since the introduction of cisplatin in cancer therapy, metal complexes and organometallic compounds have been gaining importance in oncology (Panchal 1998; Sanna et al. 2002). Metal-based compounds increase the possibility of developing molecules better-suited for binding to specific biological targets (Sanna et al. 2002; Hartinger et al. 2006).

Ruthenium complexes appear particularly promising; although they exhibit lower cytotoxicity as compared to cisplatin, they are better tolerated in vivo (Sanna et al. 2002; Hartinger et al. 2006; Menezes et al. 2007). Research on bioactive ruthenium(II) complexes is very active (Chen and Wong 2008). These studies have led to the development of ruthenium based anticancer agents (Yan et al. 2005; Ang and Dyson 2006; Hartinger et al. 2006). Research groups of Sadler, Dyson, Keppler and Reedijk have synthesized a remarkably large number of Ru(II)/Ru(III) organometallic complexes that are being tested for anticancer activity. Ruthenium complexes are slitly cytotoxic but do not affect normal cells significantly. Ruthenium drugs are very promising candidates for novel cancer therapy, with two drugs already in clinical trials, NAMI-A and KP1019 (Cocchietto and Sava 2000; Zorzet et al. 2001; Hartinger et al. 2006). Thus, there is growing interest in the use of new organometallics for the treatment of various cancers and the development of safer and more effective therapeutic agents (Panchal 1998; Silveira-Lacerda et al. 2009). In the present work, we studied the cytotoxic activity of *cis*-tetraammine(oxalato)ruthenium(III) dithionate cytotoxicity towards human chronic myelogenous leukemia (K-562) tumor cell line.

The antiproliferative activity of *cis*-tetraammine(oxalato)ruthenium(III) dithionate, a ruthenium-based complex, have been evaluated *in vitro* against human leukemia (K-562) cells using trypan blue and MTT assay. Inhibition of cell proliferation is an important potency indicator for chemotherapeutic drugs. As shown in Figures 1 and 2a and b, the tested compound induces cell death in a dose and time dependent manner on K-562 cells. It is found that the effect was improved linearly while prolonging the incubation time. The determined IC_50_ values of this complex, 18.28 μM (Figure 2a), is considerably the same of those of the commercially used antineoplastic drugs cisplatin (IC_50_ = 11 μM) and oxaliplatin (IC_50_ = 18 μM) on the same tumor cell line (Štarha et al. 2009). These results corroborate previous observations that r(III) complexes induces cytotoxicity towards tumor cells such as human Jurkat, HeLa and SK-BR-3, and murine S-180 and A-20 tumor cell lines (Frasca et al. 2001; Silveira-Lacerda et al. 2009).

For ruthenium(II) complexes as methylimidazole (RMC1) he also found having cytotoxicity of 17.34 mg mL^-1^ for A549, 18.89 mg mL^-1^ for A375 and 20.25 mg mL^-1^ for Hep G2, respectively. The same compound exhibits cytotoxicity of 51.59 mg mL^-1^ for HBE (basal lineage), as well as demonstrating that the compound RMC1 ruthenium II (Yang et al. 2012). The complex [Ru(phen)_2_(ƥ-MOPIP)]^2+^ can effectively inhibit proliferation of the A375 cell line with a low IC_50_ (5.9 ± 1.1 mM). [Ru(bpy)_2_(dppn)]^2+^ exhibits high cytotoxicity against human HT-29 and MCF-7 cancer cell lines comparable to that of cisplatin induces cell death in a dose and time dependent manner (Schatzschneider et al. 2008), and [Ru(dmp)_2_(DBHIP)]^2+^ can effectively induce apoptosis of the BEL-7402 cell line (Liu et al. 2010).

The lower general toxicity of ruthenium compounds compared to platinum drugs has been attributed to the ability of ruthenium compounds to specifically accumulate in cancer tissues. The higher specificity of these compounds for their targets may also be linked to their selective uptake by the tumor compared with healthy tissue and to selective activation by reduction to cytotoxic species within the tumor (Bergamo et al. 1999; Allardyce et al. 2003; Clarke 2003).

Ruthenium-chloro complexes tend to undergo hydrolysis in aqueous media leading to the generation of cationic Ru–OH_2_ complexes capable of reacting with DNA with greater ease than the corresponding chloro complexes (Melchart et al. 2007; Bacac et al. 2004; Hotze et al. 2004). The hydrolyzed complexes interact with the N7 of guanine in DNA duplexes leading to disruption of the structure of genetic material (Chen et al. 2003).

To explore the mechanisms of the cytotoxic effects produced by *cis*-tetraammine(oxalato)Ruthenium(III) dithionate, drug-induced changes in cell cycle distribution were examined. Compared to the cell cycle profiles of untreated cells, flow cytometric analysis indicated the sub-G1 arresting effect of ruthenium compound on K-562 cells, as ruthenium(III) dithionate already induced a 48.2% increase in the number of sub-G0/G1 cells after a 48 h incubation. The ruthenium(III) compound caused a minor S phase accumulation after incubation at concentrations of 40 and 150 μM for all periods of exposition Figure 4. Treatment with 40 μM of *cis*-[Ru(C_2_O_4_)(NH_3_)_4_]_2_(S_2_O_6_) induced respectively a 1.7-fold, 2.2-fold and 2.4-fold increase in the number of sub-G0/G1 cells when compared to control for 24, 48 and 72 h, respectively Table 1.

Furthermore, another important biochemical sign of apoptosis was studied: DNA laddering and fragmentation (data not shown), possibly representing DNA cleavage into oligonucleosomal fragments through activation of the cysteine protease caspase-3, which is involved in the proteolytic cleavage of key downstream proteins such as poly (ADP-ribose) polymerase (PARP) (Bortner et al. 1995; Clarke and Stubbs 1996). This process ultimately results in DNA fragmentation and apoptotic death (Bortner et al. 1995). Although this type of binding has been suggested to mimic, to some extent, cisplatin-induced DNA crosslinking, other types of damage may exist that dominate or contribute to the biological activity of this drug prototype.

Several studies have been using this test to corroborate the results obtained in other assays. In the present study, results derived from DNA laddering assay did not show K-562 DNA fragmentation, instead the results shows DNA integrity and even differences on DNA content. Is important note that this data does not corroborate with more sensitive techniques such as flow cytometry and comet assay, used in this work. These techniques confirmed that the ruthenium(III) compound interfere on cell cycle and induces cells to enter apoptosis. Thus, we can infer that the technique of DNA gel electrophoresis is not best assay to assess the degradation of genome DNA.

The present study also evaluated the DNA-damaging effects detected by the alkaline version of the comet assay in K-562 cancer cells. This assay has been used as a test to predict the risk to develop certain diseases (renal cell carcinoma, cancers of the bladder, esophagus, and lung) due to susceptibility of the individual to DNA damage (Ribeiro et al. 2009). The *in vitro* comet assay is proposed as an alternative to cytogenetic assays in early genotoxicity/photogenotoxicity screening of drug candidates as well as for neurotoxicity (Witte et al. 2007).

The alkaline comet assay has been used to assess the genotoxicity of chemicals, environmental exposures to carcinogens, toxins, and physical agents both *in vitro* and in vivo (Trzeciak et al. 2000; Sekihashi et al. 2002). This method was also used to measure DNA repair capacity in live cells (Banath et al. 1998) and acellular systems (Dusinská et al. 2004).

In our study, *cis*-[Ru(C_2_O_4_)(NH_3_)_4_]_2_(S_2_O_6_) shows a significant increase in tailed cells in any of the concentrations tested compared with negative control (Figure 3). Consequently, the concentration of *cis*-[Ru(C_2_O_4_)(NH_3_)_4_]_2_(S_2_O_6_) might be associated cytotoxicity with direct effect on K-562 cells DNA. Thus, it can be deducted that ruthenium-based compounds present selectivity to enter both tumor and normal cells. It is known that all the body cells present transferrin receptors, particularly tumor cells, in which these receptors are found in higher numbers. Due to these features, higher quantities of ruthenium complexes penetrate tumor tissue, reduce ruthenium(III) to ruthenium(II) and binding to DNA (guanine), and promote strand breaks (Clarke and Stubbs 1996; Lebwohl and Canetta 1998; Sava and Bergamo 2000; Katsaros and Anagnostopoulou 2002; Galanski et al. 2003; Desoize 2004). Another hypothesis is that even in small quantities this compound might enter normal cells, because ruthenium(III) complexes are activated only by its reduction when it finds an environment with high concentration of glutathione, low pH, and occurrence of hypoxia. It is very common to find tumor cells presenting these conditions since it is known that these cells have high levels of glutathione and oxygen consumption when the nutrients are quickly used due to their accelerated development promoting hypoxia (Sava and Bergamo 2000). Consequently, these cells need more glycolitic energy, which increases the level of lactic acid in the tissues and causes a decrease in the media pH. It is worth emphasizing that ruthenium(III) compounds can be used as pro-drugs that are activated by in vivo reduction to ruthenium(II) (Desoize 2004; Sava and Bergamo 2000).

Broadening the chemotherapeutic arsenal depends on understanding existing agents with a view toward developing new modes of attack. Indeed, few of the organometallics compounds may function in a manner analogous to cisplatin, which appears to bend DNA by cross-linking adjacent guanines, thereby causing a class of DNA binding proteins to adhere to the site. Overall, the broad class of ruthenium(III) antitumor agents appears to differ from cisplatin by favoring interstrand rather than intrastrand cross-links (Clarke et al. 1999). In agreement with this hypothesis, it was demonstrated that NAMI-A induced apoptosis in the ECV304 transformed human endothelial and KP1019 in colorectal carcinoma cell lines via activation of caspase-3 and DNA fragmentation (Bortner et al. 1995; Nunez et al. 1998; Kapitza et al. 2005).

Despite the resounding success of cisplatin and closely related platinum antitumor agents, the movement of other transition-metal antitumor agents toward the clinic has been exceptionally slow (Clarke et al. 1999). Non-Platinum chemotherapeutic metallopharmaceuticals hold much promise for the future, and needs to be actively explored in a large variety of tumor types in combination therapies. Besides the already well established NAMI-A and KP-1019 activities on gastrointestinal, breast, prostate, and ovarian cancers (Pluim et al. 2004; Bergamo and Sava 2007), development as a potential anti-tumor drug.

## 4 Conclusions

The present results indicate that *cis*-[Ru(C_2_O_4_)(NH_3_)_4_]_2_(S_2_O_6_) is worthy of further development as a potential anti-tumor drug that could be used in the treatment of leukemia. Thus, additional studies are needed to determine the molecular mechanisms of the active components and to evaluate the potential in vivo anticancer activity of the *cis*-tetraammine(oxalato)ruthenium(III) dithionate.

## 5 Material and methods

### 5.1 Synthesis of *cis*-[Ru(C_2_O_4_)(NH_3_)_4_]_2_(S_2_O_6_)

**Figure 5:**
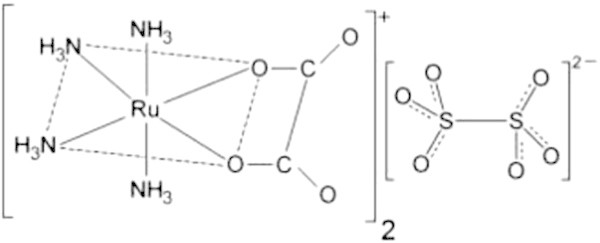
**Chemical structure of** ***cis*** **-tetraammine(oxalato)ruthenium(III) dithionate.**

The *cis*-[Ru(C_2_O_4_)(NH_3_)_4_]_2_(S_2_O_6_) complex (Figure 5), was synthesized at the Universidade Federal de Uberlândia (UFU, Minas Gerais, Brazil) following a standard protocol described by Pereira et al. ( 2009). The compound was characterized by electronic spectra at room temperature with the HP 8453 spectrophotometer with diodes arrangement, interfacing a compatible PC HP Vectra XM, using quartz cells. Carbon and hydrogen microanalyses were performed by the staff of the Analitical Centre of the Chemistry Institute of Universidade de São Paulo (USP, São Paulo, Brazil).

### 5.2 Cell culture

The human chronic myelogenous leukemia (K-562) cell line (ATCC number CCL-243™) was obtained from the American Type Culture Collection (ATCC, Rockville, MD, USA). The cells were cultured in RPMI 1640 medium (pH 7.2–7.4) supplemented with 100U mL^-1^ penicillin G, 100 μg mL^-1^ streptomycin, 2 mM L-glutamine, 1.5 g L^-1^ sodium bicarbonate, 4.5 g L^-1^ glucose, 10 m*M* HEPES, 1.0 m*M* sodium pyruvate and 10% fetal calf serum (FCS) (all reagents were obtained from Gibco, Grand Island, NY, USA) at 37°C, 5% CO_2_ and humidified atmosphere.

The cells were disposed into 96 well plates (1 × 10^5^ cells/well) and cultured in RPMI 1640 medium. Cells were harvested at specified intervals and the number of cells per well was determined by cell counting with a hemocytometer (Neubauer chamber). Briefly, tumor cells were aspirated, washed in sterile PBS and an aliquot of the cell suspension was put in Trypan Blue 1% (m/v) (Sigma-Aldrich, St. Louis, MO, USA) and counted. Only cell dilutions with > 95% of viable cells were included in the posteriors analysis.

### 5.3 Cell citotoxicity (Trypan blue staining)

The citotoxicity of the K-562 cells was evaluated by the trypan blue exclusion assay. The tumor cells were incubated for 24 h, 48 h and 72 h with different concentrations of the tested ruthenium compound *cis*-[Ru(C_2_O_4_)(NH_3_)_4_]_2_(S_2_O_6_) (from 0.015–32 μM) at 37°C. Additionally, Carboplatin (50 μM) and Paclitaxel (25 μM) were applied as positive control. After incubation, the cells were washed in PBS (pH 7.4) and suspended in a complete RPMI 1640 medium. Then 40 μL of the trypan blue solution (0.4%, Sigma) and 10 μL of the cell suspension were mixed and after 5 min the percentage of viable K-562 cells was evaluated under brightfield optical microscope using a newbauer chamber. The correlation between the viable cells (that excluded trypan blue dye) and dead cells (stained cells) were assessed. The results are presented as mean ± S.D. (standard deviation) from three independent experiments.

### 5.4 Viability assay

Cytotoxic activity of *cis*-[Ru(C_2_O_4_)(NH_3_)_4_]_2_(S_2_O_6_) on K-562 was measured by modified MTT assay (Mosmann 1983), which is based on the reduction of yellow 3-(4,5-dimethylthiazol-2-yl)-2,5-diphenyl tetrazolium bromide (MTT) to dark-blue, insoluble formazan in mitochondria of the living cells. The MTT assay was performed in 96-well tissue culture plates (Nalge-Nunc, Rochester, NY, USA) as follows: the cells (1 × 10^5^/well) were seeded in tissue culture plates and incubated with the different concentrations of ruthenium compound (from 0.15–150 μM) dissolved in a total volume of 100 μL/well for 24 h and 48 h at 37°C and 5% CO_2_. The cells incubated with medium only served as a control. Following the exposure to the tested substances, the cells were incubated with 10 μL of MTT solution (5 mg mL^-1^) (Sigma-Aldrich, St. Louis, MO, USA) for 3 h. The microplates were then centrifuged (300 × *g*/15 min/10°C) and the culture media were discarded. Afterwards 200 μl of PBS/20% of SDS (lauryl sulfate) solution (Sigma-Aldrich, USA) was added to each well to stop the reaction and plates were kept in the dark overnight. After the next 12 h, the absorbance of dissolved formazan was measured by a Stat Fax 2100 microplate reader (Awareness Technology, Palm City, FL, USA) at 565 nm. The cell viability was expressed in% related to control (100% of viability). The cytotoxic rate was calculated as follows: cytotoxicity (%) = [1 – (absorbance of the treated wells) / (absorbance of the control wells)] × 100%. The cytotoxic effect of the tested substances was determined in at least three independent experiments, where each one of the culture plates contained the wells with tested concentration in triplicate, the wells with control (cells in medium) and the blank (culture medium alone). The 50% inhibitory concentration (IC_50_) value was determined using GraphPad Prism 4.02 for Windows (GraphPad Software, San Diego, CA, USA).

### 5.5 DNA laddering assay

Briefly, 2 × 10^6^ cells (K-562) were treated with different concentrations of *cis*-[Ru(C_2_O_4_)(NH_3_)_4_]_2_(S_2_O_6_) (from 0.15–150 μM) for 24 h at 37°C and 5% CO^2^. Cells were harvested and centrifuged at 300 × *g*/15 min/10°C and washed with PBS. The cells were then ressuspended at a concentration of 1 × 10^6^ cells mL^-1^ in an extraction buffer (1 mol Tris–HCl, 2 mol Na_2_EDTA, 0,5 g m L^-1^ SDS) and treated with 20 mg L^-1^ RNase A at 37°C for 60 min, followed by incubation with proteinase K (100 mg L^-1^) at 37°C for 60 min. An equal volume of saline solution (NaCl 6 M) was added to the cells and centrifuged at 13,000 × *g* for 10 min. The supernatant was collected and 2 volume ethanol (-20°C) were added. The samples were centrifuged at 13,000 × *g* for 30 min at 4°C. The supernatant was then discarded and the pellets dissolved in TE buffer (1×). The concentration of DNA was detected using a UV spectrophotometer (Beckman DU-640, USA). The DNA (5 μg/tube) was transferred to a 1.5% agarose horizontal gel, and electrophoresis was performed at 100 V cm^-1^ for 90 min. The DNA in the gels was visualized by ultraviolet transillumination after staining with ethidium bromide (5 μg mL^-1^) using an Omega® molecular imaging system (UltraLum Inc., Claremont, CA, USA).

### 5.6 Comet assay

An aliquot of from each K-562 culture was taken after 24 and 48 h incubation for the alkaline version of the comet assay (Ribeiro et al. 2009). The compound *cis*-[Ru(C_2_O_4_)(NH_3_)_4_]_2_(S_2_O_6_) was studied at different concentrations (from 10–150 μM). Briefly, 300 μL of the cell suspension was centrifuged for 5 min (500 rpm) in a refrigerated microcentrifuge (Sorvall Legend Mach 1.6 R, Thermo Fisher Scientific, Waltham, MA, USA). The resulting pellet was homogenized with 80 μL of a low melting point agarose (0.5%), spread onto microscope slides pre-coated with a normal melting point agarose (1.5%), and covered with a cover slip. After 5 min at 4°C, the cover slip was removed, and the slides were immersed in cold lysis solution [2.4 *M* NaCl, 100 mM ethylenediamine tetraacetic acid (EDTA), 10 mM Tris, 10% DMSO, and 1% Triton-X, pH 10] for 24 h.

After lysis, the slides were placed in an electrophoresis chamber and covered with electrophoresis buffer (300 mM NaOH per 1 mM EDTA, pH > 13) for a remaining 20 min to allow DNA unwinding. The electrophoresis proceeded for 20 min (25 V and 300 mA). Afterwards, the slides were submerged for 15 min in a neutralization buffer (0.4 M Tris– HCl, pH 7.5), dried at room temperature, and fixed in 100% ethanol for 5 min. All the steps were conducted in the dark to prevent additional DNA damage. Slide staining was performed immediately before analysis using ethidium bromide (20 μg mL^-1^). Slides were prepared in duplicate, and 100 cells were screened per sample (50 cells from each slide) in a fluorescent microscope (Leica Microsystems GmbH, Wetzlar, Germany) equipped with an excitation filter of 515–560 nm and a barrier filter of 590 nm using a × 40 objective. The analyse nucleus was realized in image analysis system software CometScore 15 according to the migration of the fragments, as previously proposed as follows: class 0 (no damage); class 1 (little damage with a short tail length smaller than the diameter of the nucleus); class 2 (medium damage with a tail length one or two times the diameter of the nucleus); class 3 (significant damage with a tail length between two and a half to three times the diameter of the nucleus); and class 4 (significant damage with a long tail of damage greater than three times the diameter of the nucleus). A value (damage index, DI) was assigned to each comet according to its class, according to the following formula:

DI = (0 × n_0_) + (1 × n_1_) + (2 × n_2_) + (3 × n_3_) + (4 × n_4_), where n = number of cells in each class analysed.

DI thus ranged from 0 (completely undamaged: 100 cells × 0) to 400 (with maximum damage; 100 cells × 4).

### 5.7 Cell cycle analysis by flow cytometry

In order to investigate the possible effect of the ruthenium compound on cell cycle progression, K-562 cells were treated with different concentrations of *cis*-[Ru(C_2_O_4_)(NH_3_)_4_]_2_(S_2_O_6_) (from 1.5–150 μM) for 24, 28 and 72 h. Briefly, 5 × 10^5^ cells were harvested by centrifugation, washed with PBS, fixed with 70% (v/v) cold aqueous ethanol and stored overnight at -20°C. The fixed cells were washed with PBS and incubated with propidium iodide (PI; Sigma-Aldrich, St. Louis, MO, USA) containing 0.05% RNase. Samples were incubated at 4°C in the dark and analyzed by flow cytometry (FACS CantoII, BD Biosciences, San José, CA, USA). The percentage of cells in G1, S, G2 and sub-G1 was analyzed using ModFit LT software (Verity Software House, Topsham, ME, USA).

### 5.8 Statistical analysis

Three independent *in vitro* experiments were carried out. Statistical results were expressed as the mean ± standard deviation of the means obtained from triplicates of each independent experiment. Correlation tests were performed to determine the effects of concentration of ruthenium complex on tumor cell lines. Statistical significance of differences *(p < 0.05) as compared to untreated cells (control) was evaluated by applying analysis of variance (ANOVA) and Tukey or Dunnet’s post tests, when applicable. The IC_50_ (concentration that produces a 50% inhibitory effect on the evaluated parameter) was graphically obtained from the dose–response curves.

### Ethical Approval

No studies involving humans or experimental animals were conducted in this work. The human chronic myelogenous leukemia (K-562) cells were purchased from the American Type Culture Collection (ATCC, Rockville, MD, USA) and cultured in vitro.
